# The relationship between cognitive and global function in patients with schizophrenia and mood disorders: a transdiagnostic network analysis

**DOI:** 10.3389/fpsyt.2025.1643369

**Published:** 2025-07-30

**Authors:** Yuanyuan Zhu, Rongrong Zhang, Longyan Ni, Zhaoyang Xie, Shuiping Lu, Shiping Xie, Xiangrong Zhang

**Affiliations:** ^1^ Department of Psychiatry, Affiliated Brain Hospital of Nanjing Medical University, Nanjing, China; ^2^ Department of Psychology and Cognitive Science, East China Normal University, Shanghai, China; ^3^ Department of Geriatric Psychiatry, Affiliated Brain Hospital of Nanjing Medical University, Nanjing, China

**Keywords:** neurocognition, social cognition, global function, network analysis, intervention target

## Abstract

**Objective:**

Cognitive deficits present transdiagnostic characteristic and partly explain the poor functional outcomes of patients with mental disorders. Understanding the relationships between neurocognition, social cognition, and global function may help identify new cognitive intervention targets. We aimed to model the complex interrelationships among these variables with Gaussian Graphical Modeling in a transdiagnostic sample.

**Methods:**

A total of 482 individuals were included in this study, comprising 281 patients with first-episode schizophrenia, 128 patients with bipolar disorder, and 73 patients with major depressive disorder. The Wechsler Adult Intelligence Scale, the MATRICS Consensus Cognitive Battery, and the Global Assessment of Functioning Scale were evaluated. The interaction and centrality indexes of cognitive and global function were analyzed by network analysis.

**Results:**

In the transdiagnostic network, speed of processing (SOP) and verbal learning (Vrbl) exhibited higher centrality indexes. The cognitive nodes closely associated with global function included working memory (WM), and attention/vigilance (AV). When subjects were modeled separately by gender, no significant differences were found between males and females.

**Conclusion:**

The close connections between WM, AV, and global function as well as the high centrality indexes of SOP and Vrbl suggest that these domains share aspects of pathophysiology in schizophrenia and mood disorder. However, the data-driven approach limited our interpretation of the results. Theory-driven model should be further validated to elucidate causal pathways and find more promising approaches to recovery.

## Introduction

Previous studies have consistently shown that individuals with mental disorders often experience cognitive deficits to varying degrees. These deficits are not specific to any one disorder but rather exhibit transdiagnostic characteristic. This perspective has been further corroborated by subsequent studies (1–3). Notably, a large meta-analysis conducted in 2021 analyzed executive function, working memory (WM), and speed of processing (SOP) across 12 mental disorders. The study not only proposed but also validated the C-factor hypothesis, which underscores the transdiagnostic nature of cognitive impairment (4).

The clinical remission rates of patients with schizophrenia and depression after 1 year were 39.2% and 42.3%, while the function recovery were only 17% and 38.1% (5). An array of variables such as untreated duration, simultaneous remission of positive and negative symptoms, and most cognitive variables have been pointed as important dimensions impacting the functional recovery of patients with first-episode psychosis (6). It has been suggested that cognitive function may be a better predictor of global function than the severity of affective symptoms in bipolar disorder (7). Researchers (8) divided the predictors of remission into non-modifiable factors (such as sex, age) and modifiable factors (such as education, disease symptoms, work status), they found that female sex and older age were predictors of positive clinical outcomes in depression, schizophrenia spectrum, and substance use. There were many researches on gender differences in cognitive function of mental disorders. But the results so far were inconsistent, most supported that women perform better than men on certain cognitive domains (9, 10). The cognitive functions’ transdiagnostic nature and their impact on global function make it becoming one of the important targets in clinical intervention. Therefore, understanding the important distribution characteristics of cognitive domains and their relationships with global function are of great significance for future precise intervention. Previous researches had predominantly focused on exploring the characteristics of cognitive impairment (11) and comparing the extent of cognitive deficits across different mental disorders (12). However, these studies have generally failed to provide a comprehensive understanding of the relationships among the impaired cognitive domains, especially from a perspective of transdiagnostic.

The concept of transdiagnostic was firstly appeared in a report on cognitive behavioral therapy for patients with eating disorders (13), the authors suggested that anorexia nervosa, bulimia nervosa and atypical eating disorder share similar psychopathology, clinical features, disease maintenance factors, and finally proposed a new transdiagnostic cognitive behavioral therapy. Subsequently, transdiagnostic researches expanded to depression, anxiety, substance use, and schizophrenia spectrum disorders (14, 15).The transdiagnostic framework has also evolved beyond its initial focus on early psychotherapy interventions (16–18) to cross-sectional factors of disease maintenance or recurrence (19–21), clinical features (22, 23), and cognitive function (4, 24).

Network analysis can reveal the relationships among various variables more intuitively and is suitable for transdiagnostic studies because of some similar viewpoints. The network theory of psychopathology believes that mental disorders are formed by the interaction of symptoms. The mutual feedback between symptoms will play an important role in the persistence of mental disorders (25). A number of studies have used network analysis to assess the relationships between the symptoms of mental disorders in order to understand which symptoms are more important in the disease (26), which symptoms act as a bridge between different clusters (27). S. Galderisi et al. (28) studied the relationship between personal resources, environmental factors, psychopathology and real-life functions in schizophrenia with network analysis. They found that functional ability and daily living skills were at the core of the network and highly correlated, while positive symptoms in psychopathology were at the periphery of the network. Another study (29) added cognitive function dimension to the network analysis, and found that both neurocognition (NC) and social cognition (SC) were important contributors to functional outcomes, WM was particularly related to functional outcomes. Current research samples about network analysis are relatively single, few studies have conducted network analysis from a cross-diagnostic perspective. S. Fritze et al. (30) modeled the relationships between sensorimotor, cognition and global function in a transdiagnostic sample of 212 patients with mental disorders, they found sensorimotor symptoms may represent a transdiagnostic therapeutic target. However, the unbalanced diagnostic groups limited the inference of the results. Our study aimed to examine the relationships between a broader range of cognitive domains and global functioning within a larger transdiagnostic sample as well as investigate the impact of gender on these networks. Our goals can be divided into the following three points: 1. Establish the network of cognitive and global function. 2. Identify key cognitive subtypes influencing global functioning. 3. Explore the gender differences in cognitive and global function networks.

## Methods

### Study participants

A total of 482 subjects, including 281 first-episode schizophrenia, 128 bipolar disorder patient (depressive phase), and 73 major depressive disorder patients were recruited in this study. All the patients were from the outpatient and inpatient departments of Nanjing Brain Hospital, and the healthy controls were recruited from the surrounding community. Inclusion criteria for the patients were as following: Fulfilling DSM-5 criteria for bipolar disorder (depressive phase), major depressive disorder or schizophrenia. The Chinese Han population, aged 16–45, right-handed. Education years ≥ 8 years. No previous exposures to any psychotropic drugs including benzodiazepines or other physical therapy. Exclusion criteria: History of neurological disease or other major physical disease. History of alcohol or substance abuse. Intelligence quotient (IQ) < 70. The Inclusion criteria for the healthy controls were identical to those of the patients’ group, with the exception being the absence of a psychotic disorder. This study was approved by the Ethics Committee of the Affiliated Brain Hospital of Nanjing Medical University (Ethics number: 2021-KY075-01). All subjects signed informed consent, and subjects under the age of 18 were signed by their guardians.

### Clinical assessments

All assessments were completed by physicians trained in standardized neuropsychological tests on the same day as enrollment. The MATRICS Consensus Cognitive Battery (MCCB) and the Wechsler Adult Intelligence Scale (WAIS) were used to assess cognitive function and IQ. Additionally, the global assessment of functioning (GAF) was used to evaluate global function of participants. MCCB was originally recommended for assessing cognitive function in schizophrenia (31). However, recent studies have demonstrated that the MCCB also exhibits relatively high internal consistency and reliability among patients with bipolar disorder and depression (32, 33). The MCCB includes seven cognitive domains, namely, SOP, WM, attention/vigilance (AV), Vrbl, visuospatial memory (Vis), reasoning and problem solving (RPS), and SC. Higher scores in these domains indicate better cognitive functioning. For the purposes of this study, the T-scores of each cognitive subdomain were calculated and used as the final inclusion metric. The GAF is a widely used clinical scale for assessing overall functioning (34). It measures symptoms severity and functional outcomes on a scale ranging from 0 to 100, with higher scores indicating less severity of symptoms and better function outcome.

### Statistical analysis

Firstly, a descriptive analysis of demographic data was conducted with SPSS27.0. Nest, R (version 4.3.3) and R-Studio were used to perform network analysis. The qgraph package was used to estimate and visualize network. In the network, each visual item is called a node, and the lines between nodes are called edges which are also the variables we want to evaluate. The blue edge indicates a positive correlation, and the red indicates negative. The thicker of the edges, the stronger of the correlation. To avoid false-positive relationships, we used the extended Bayesian information criterion (EBIC) lasso to regularize the network (35–37), which can assign zero values to edges with lower edge weights, and create a more compact network graph. We calculated the centrality indexes of strength and expected influence. Strength centrality is the sum of absolute values of all edge weights connected to it, which can reflect the importance of a node in the whole network. The expected influence is the average value of the influence of a node on all other nodes, which reflects the degree of activation and inhibition of the node on the whole network. Then the accuracy of edge-weights and stability of centrality indexes were estimated by bootstrapping the 95% confidence intervals (CI) and case dropping bootstrapping. A narrower CI indicates a more accurate estimation of the edge weight. The correlation stability coefficient (CS-C) was used to quantify the stability of centrality indexes. We used recommended CS-C cut-off values (optimal CS-C>0.5, minimum CS-C>0.25) (29). To verify whether the model is driven by diagnostics, we repeated the network analysis in schizophrenia and mood disorders (including major depressive disorder and bipolar disorder). Finally, the Network Comparison Test package was used to analyze the differences between the networks of males and females from four aspects (the network structure invariance test, the global strength invariance test, the edge invariance test, and the centrality invariance test). *p*<0.05 was considered statistically significant.

## Results

### Clinical and demographic characteristics

Data of 482 patients were included in the analyses. The mean age and education of the entire sample were 24.49 ± 7.14 years and 13.58 ± 2.86 years. Approximately 56.8% of the sample were male. **Table 1** shows the detailed demographic and clinical characteristics as well as the skewness and kurtosis of each variable. No data-transformation procedure was performed because of the acceptable range of skewness (cutoff is 2.0) and kurtosis (cutoff is 7.0) (38). The group differences between all the variables can be seen in **Supplementary Table S1**.

**Table 1:** Clinical and demographic variables of study participants (n=482).

Variable	Mean ± SD	Skewness	Kurtosis
Age	24.49 ± 7.13	0.85	−0.09
Education	13.53 ± 2.86	−0.03	−0.35
Sex (male/female)	274/208	–	–
Antipsychotic treatment (yes/no)	0/482	–	–
SoP	40.7 ± 11.22	−0.55	0.65
AV	41.39 ± 10.56	−0.05	−0.26
WM	38.67 ± 10.38	−0.11	0.21
Vrbl	41.9 ± 11.41	−0.47	−0.29
Vis	45.42 ± 11.16	−0.78	0.32
RPS	47.16 ± 11.46	−0.47	−0.50
SC	33.57 ± 10.47	0.59	0.41
IQ	110.71 ± 13.55	−1.13	5.14
GAF	44.77 ± 11.09	0.49	−1.06

Data are mean ± standard deviation. SD standard deviation, SOP speed of processing, WM working memory, AV attention/vigilance, Vrbl verbal learning, Vis visual learning, RPS reasoning and problem solving, SC social cognition, IQ intelligence quotient, GAF Global Assessment of Functioning.

### Network analysis

**Figure 1:**
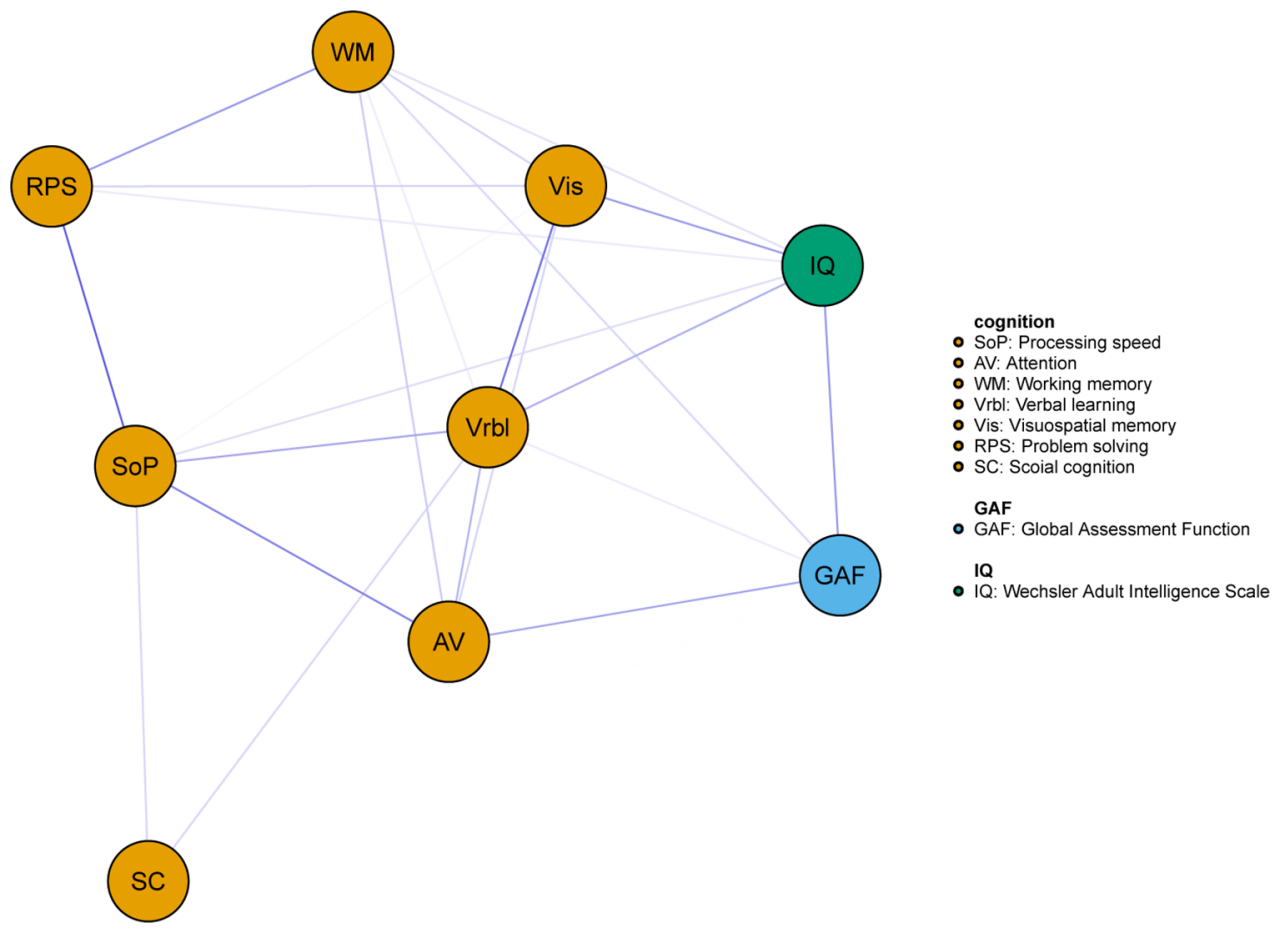
Network of the cognitive and global function for all subjects. Blue edges represent positive associations whereas red edges represent negative associations, and thickness of an edge represents the strength of association between two nodes.

As shown in **Figure 1**, the network consisted of IQ, the seven cognitive domains of MCCB, and the GAF. The GAF was positively correlated with IQ, AV, and WM, specific edge weights can be seen in **Table 2**.

**Table 2:** Partial correlation matrix of the network for all subjects.

Variable	SOP	AV	WM	Vrbl	Vis	RPS	SC	IQ	GAF
SOP	0.000	0.217	0.033	0.171	0.058	0.273	0.105	0.094	0.037
AV	0.217	0.000	0.120	0.151	0.107	0.000	0.000	0.009	0.174
WM	0.033	0.120	0.000	0.072	0.107	0.190	0.000	0.090	0.101
Vrbl	0.171	0.151	0.072	0.000	0.244	0.000	0.103	0.158	0.077
Vis	0.058	0.107	0.107	0.244	0.000	0.111	0.043	0.186	0.034
RPS	0.273	0.000	0.190	0.000	0.111	0.000	0.000	0.078	0.028
SC	0.105	0.000	0.000	0.103	0.043	0.000	0.000	0.000	−0.051
IQ	0.094	0.009	0.090	0.158	0.186	0.078	0.000	0.000	0.192
GAF	0.037	0.174	0.101	0.077	0.034	0.028	−0.051	0.192	0.000

SD, standard deviation; SOP, speed of processing; WM, working memory; AV, attention/vigilance; Vrbl, verbal learning; Vis, visual learning; RPS, reasoning and problem solving; SC, social cognition; IQ, intelligence quotient; GAF, Global Assessment of Functioning.

**Figure 2:**
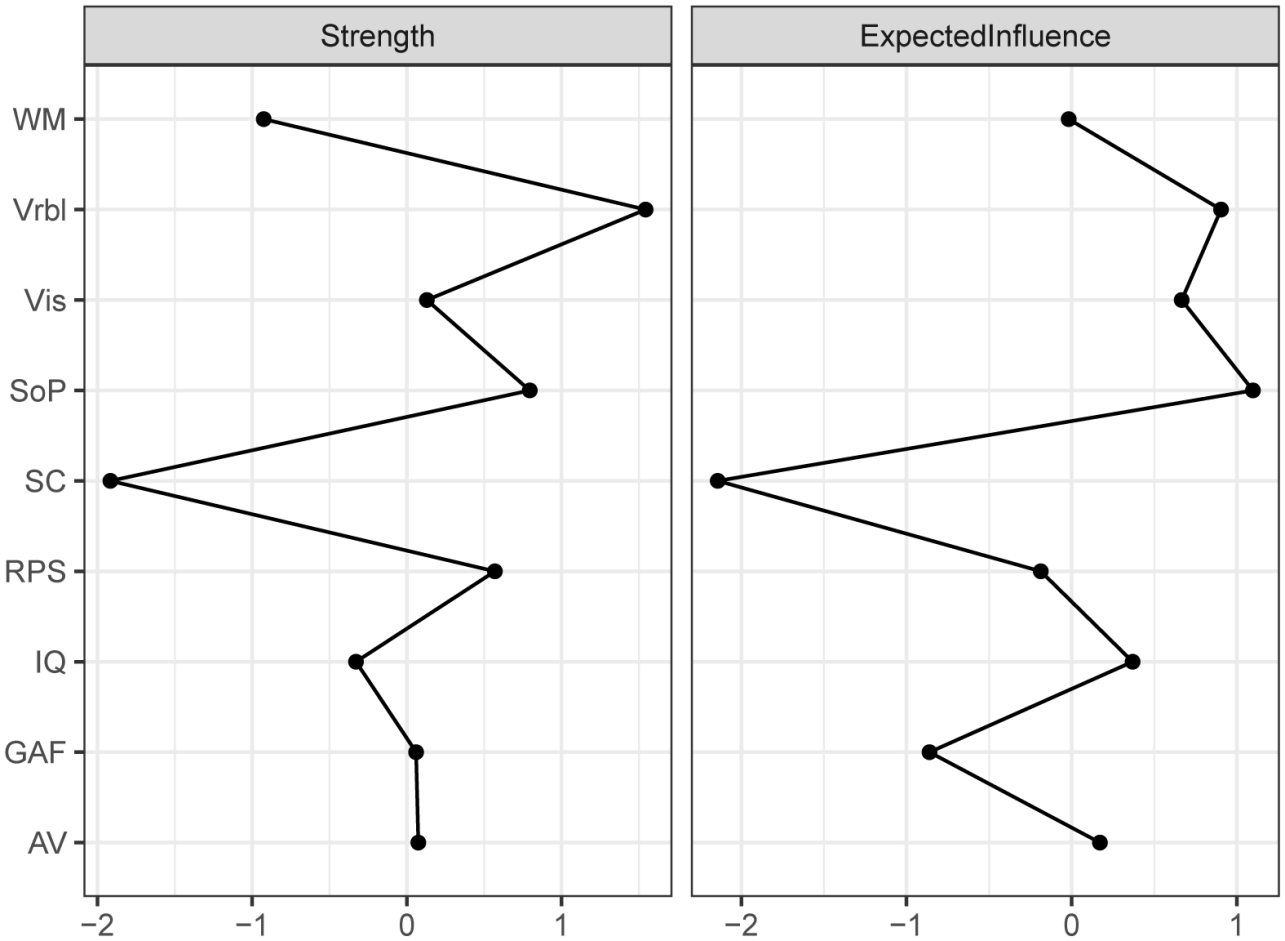
Centrality of the network for all subjects. Depicting the Strength and Expected influence of each node. WM working memory, Vrbl verbal learning, Vis visual learning, SOP speed of processing, SC social cognition, RPS reasoning and problem solving, IQ intelligence quotient, GAF Global Assessment of Functioning, AV attention/vigilance.

The centrality index of each node in the network for all subjects were shown in **Figure 2**. In this network model, the top three nodes with the strongest strength centrality were Vrbl, SOP, and RPS, while SOP, Vrbl and Vis occupied the top three positions of expected influence. SC had the lowest centrality indexes both in strength and expected influence. The estimation of the accuracy and stability of the network showed that the 95% CI value of the edge weight was relatively narrow. The CS-C of centrality indexes were as following, strength: 0.595, expected influence: 0.672. These results indicated that the overall network stability was acceptable. For details, see **Supplementary Figure S1**. When repeating the network analysis in patients with schizophrenia (n=281) and mood disorders (including major depressive disorder and bipolar disorder, n=201), only the network of schizophrenia (**Supplementary Figure S7**) was stable. The SOP and Vrbl had the highest expected influence and strength centrality index respectively (**Supplementary Figure S10**). The network results of mood disorder were not shown here due to instability.

**Figure 3:**
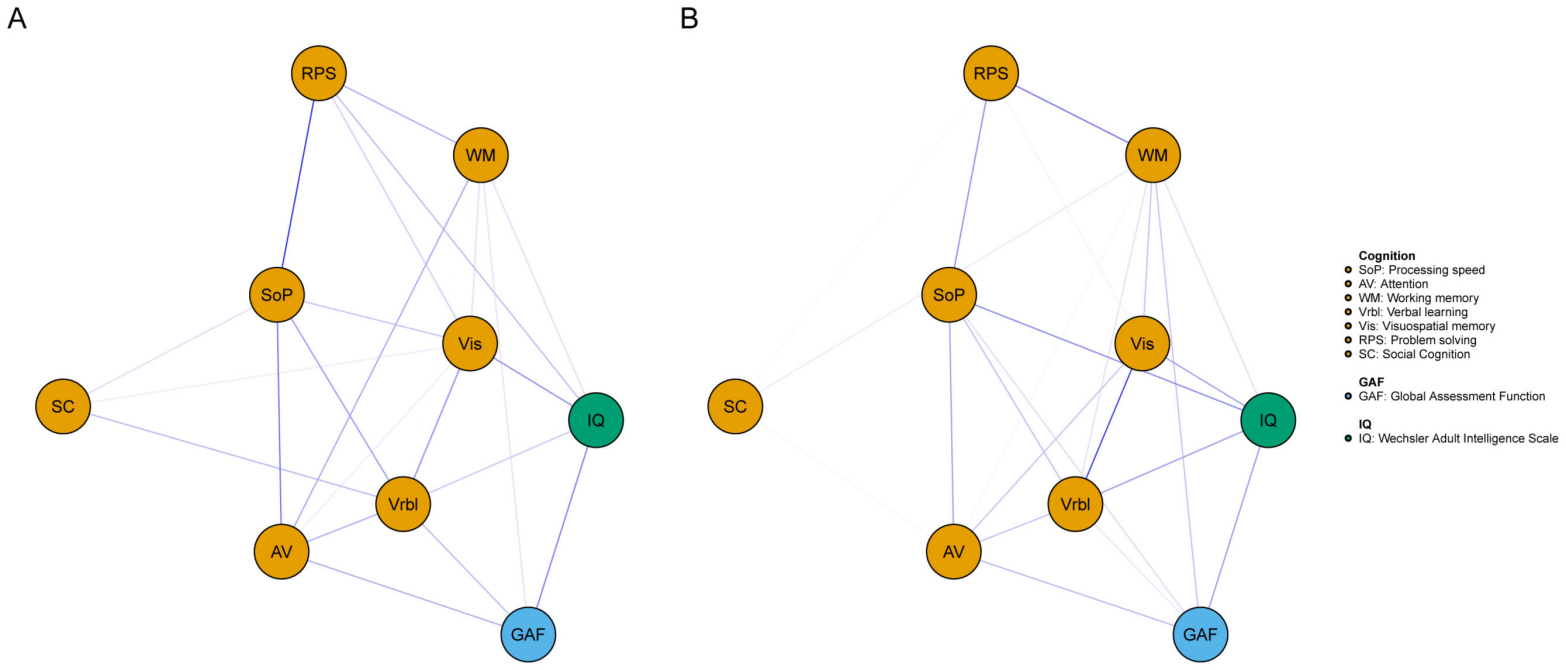
Network of cognitive and global function for different genders. A= male, B= female. Blue edges represent positive associations whereas red edges represent negative associations, and thickness of an edge represents the strength of association between two nodes.

**Figure 4:**
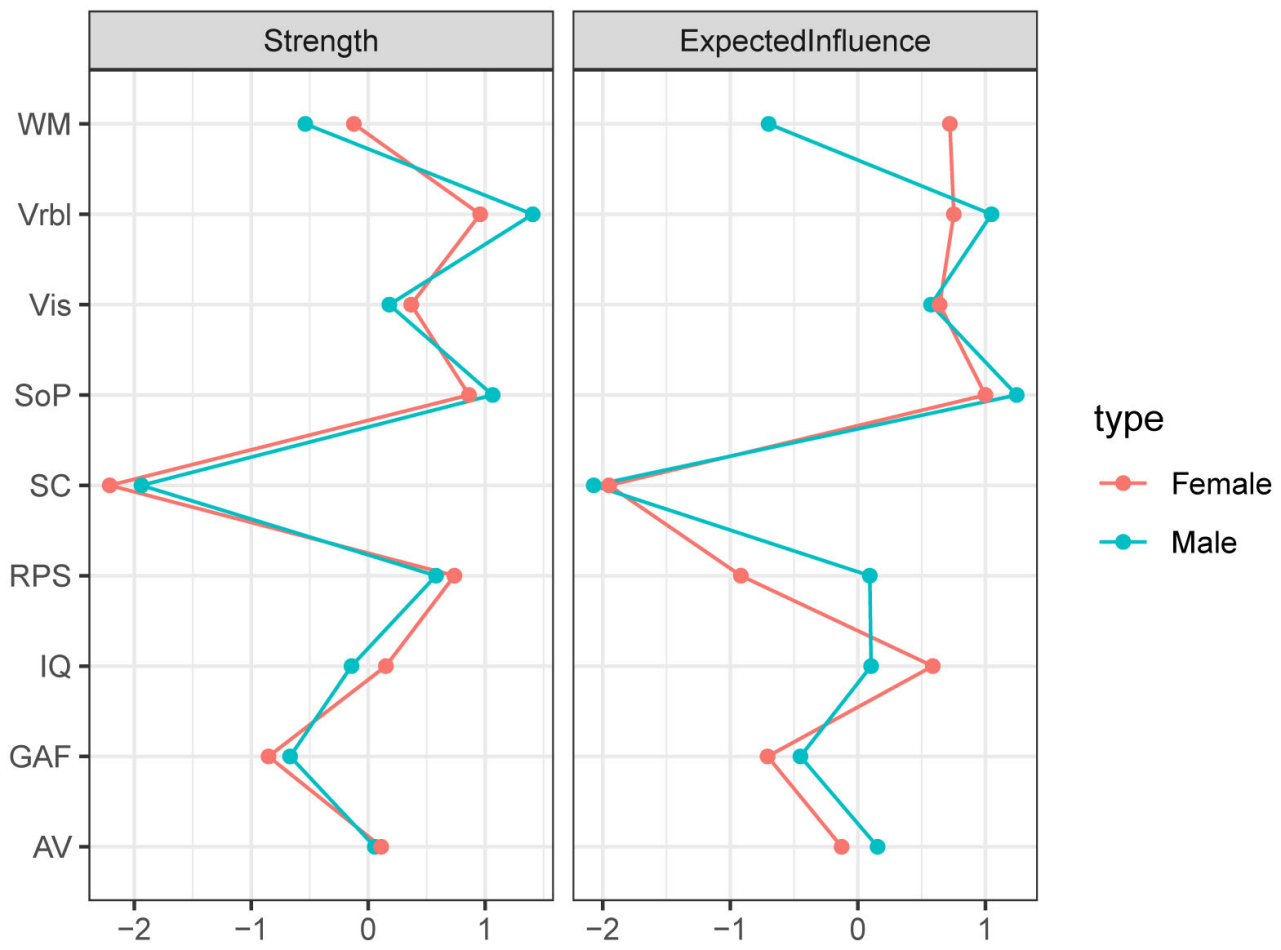
Centrality of the network for different genders. Depicting the Strength and Expected influence of each node, with red nodes representing female and green nodes representing male.

The networks for different genders were constructed respectively, then the accuracy and stability of the two networks were estimated. The CS-C of centrality indexes in the males were as following, strength: 0.518, expected influence: 0.595. While females were 0.438 and 0.438. Both the strength and expected influence indexes were within the acceptable range, details can be found in **Supplementary Figure S3** and **Supplementary Figure S5**. As we can see in **Figure 3**, the global function in the male network was positively correlated with IQ (edge weight: 0.223), AV (0.162), and Vrbl (0.142). For the females, the nodes with stronger correlation are IQ (0.186), AV (0.145), and WM (0.132). When comparing the network structures of men and women, no significant differences were found. The centrality index of each node in the male and female networks can be found in **Figure 4**.

## Discussion

In this study, we used network analysis to investigate the interrelationships between various cognitive domains and global function as well as explore significant differences in transdiagnostic networks between males and females. The results showed that SOP and Vrbl had relatively higher centrality indexes both in strength and expected influence across all networks, while the centrality indexes of SC were the lowest.

SOP involves a series of mental processes such as the acquisition, encoding and storage of input information. Consistent with several previous studies (11, 39), we also utilized the Trail Making Test, Symbol Coding, and Category Fluency from the MCCB as measures of information processing speed. While SOP is considered a limited cognitive resource for learning and memory (40), some researchers have demonstrated that it can account for the individual differences in Vrbl of older adults to some extent (41). A study examining the rates of cognitive impairment across different psychiatric disorders found that the cognitive tests with higher impairment in clinical groups mainly involved SOP, Vrbl, WM, and verbal fluency (42).The deficits of SOP and Vrbl usually have higher effect sizes in people with mental disorders (11, 43),and are related to functional outcomes (44, 45). M. Lindgren et al. (46) conducted a follow-up study and found that cognitive function, especially the SOP and SC at baseline can predict outcomes one year later in patients with first-episode psychosis, the authors suggested that both SOP and SC should be considered primary cognitive targets for clinical intervention. Our results further support the potential value of SOP and Vrbl as transdiagnostic targets for cognitive intervention in terms of the significance of the network structure.

Cognitive deficits are common among individuals with mental disorders (47, 48), the relationship between NC, SC and global function has always been a clinical concern. In our results, the node of SC was at a relatively marginal position in the network. The remaining six nodes of NC and IQ were more closely connected and exhibited stronger correlations with global function. SC appeared to influence global function indirectly through certain NC variables, such as SOP and Vrbl. These findings align with the structural view of previous studies, which suggest that NC and SC are related but largely independent constructs (49, 50). The processing of social cognition is typically predicated on the foundation of neurocognitive abilities. Only by focusing on individuals’ language, expressions, actions, and other details, can one integrate numerous pieces of information and feed them back to the brain to make corresponding judgments and inferences, which is a manifestation of SC. However, some researchers have argued that SC may be a stronger predictor of functional outcomes in schizophrenia spectrum disorders than NC, which acts as a mediating factor between functional outcomes and NC (51). Recently, network analysis has been employed to investigate the relationships between NC, SC, clinical symptoms, and functional outcomes in a sample of 408 patients with schizophrenia spectrum disorders. The results indicated that both SC and NC contribute to functional outcomes, with social skills and functional ability potentially serving as bridges connecting NC, SC, and functional outcomes (29). In our results, the correlations between SC and global function were so low in all networks that they were less evident in the network diagram. These are inconsistent with those of previous studies. As we all know, the SC includes emotional processing, theory of mind, social perception, social knowledge and attribution bias (52). The SC assessment in the MCCB primarily focuses on emotional processing. Functional outcomes, on the other hand, have been divided into four categories based on different measurements: community function, social behavior in the environment, social problem-solving, and social skills (50, 51). The authors have even called for considering the type of functional outcome when planning intervention measures in research. In our study, we used relatively single tools to measure SC and global function due to data-driven approach, so there are fewer nodes representing these two clusters. This may partly explain the inconsistencies with previous studies. Besides, the heterogeneity of assessment tools and different research methods may also contribute the inconsistency.

The study of gender differences may be a window into the pathogenesis of some mental disorders, and could help develop specific treatment strategies for these disorders. Consistent with prior research (53), we did not find gender differences in the network of cognitive and global function in terms of global node strength and edge weights. The network theory of psychopathology (25) pointed that a strongly connected network tends to transition to a disordered state when disturbed by an external field, while a weakly connected network has more flexibility and can quickly recover to a stable state when disturbed. Based on this theory, we speculate that the same external factors may have little different effect on the cognitive and global functional networks for different genders. However, as mentioned in network theory, external factors not only include changes or stress in some social environment, but also those from the body, such as inflammation and changes in specific brain areas. This underscores the need to incorporate a broader range of research variables into future network analysis studies to identify more therapeutic targets.

The primary strength of our study lies in the integration of the transdiagnostic concept with network analysis methodology. Besides, the influence of medications on cognition was minimized, as none of the participants had received antipsychotic drugs or other sleep-promoting medications before enrollment.

Through these approaches, our study displayed the relatively important cognitive nodes of patients with mental disorders, revealed the relationships between NC, SC and global function, and compared the differences of networks with different genders. It may provide us some ideas for understanding the cognitive network structures of different genders and selecting transdiagnostic cognitive intervention targets. Of course, there are still some limitations in this study. First of all, the design of cross-sectional research will hinder our judgment on the causal relationships and the direction of influence between cognitive nodes. Second, although we used well-validated tasks to measure cognitive, the data-driven approach limited the number of nodes for SC and global function, and failed to take into account other factors affecting global function, such as psychopathology, family environment, and general clinical information. Therefore, our results should be interpreted with caution. The theory-driven model should be further validated in the future. In addition, further intervention studies combining longitudinal design, imaging or electroencephalogram research tools and network analysis are needed to elucidate causal pathways and find more promising approaches to recovery.

## Conclusion

The close connections between WM, AV, and global function as well as the high centrality indexes of SOP and Vrbl suggest that these domains share aspects of pathophysiology in schizophrenia and mood disorder. However, the data-driven approach limited our interpretation of the results. Theory-driven model should be further validated to elucidate causal pathways and find more promising approaches to recovery.

## Data Availability

The raw data supporting the conclusions of this article will be made available by the authors, without undue reservation.
