# Supplementary material for: The relationship between cognitive and global function in patients with schizophrenia and mood disorders: a transdiagnostic network analysis
Source: Front Psychiatry. 2025 Jul 30;16:1643369. doi: 10.3389/fpsyt.2025.1643369 (PMC12344734; doi:10.3389/fpsyt.2025.1643369)
Supplement: Supplementary file 1 [file Supplementaryfile1.docx]

Supplementary Material

# Supplementary Tables

| Table S1. Clinical and demographic variables of SCH and MOD participants | | | | |
| --- | --- | --- | --- | --- |
| Variable | SCH(n=281) | MOD(n=201) | t/X*^2^* | *p* |
| Age | 24.51±7.32 | 24.46±6.89 | 0.070 | 0.944 |
| Gender(m/f) | 197/84 | 77/124 | 48.300 | <0.001 |
| EDU | 13.04±2.90 | 14.23±2.66 | ﹣4.610 | <0.001 |
| SOP | 38.41±11.57 | 43.89±9.89 | ﹣5.440 | <0.001 |
| AV | 38.74±10.57 | 45.08±9.41 | ﹣6.789 | <0.001 |
| WM | 36.17±10.13 | 42.17±9.71 | ﹣6.520 | <0.001 |
| Vrbl | 39.08±11.71 | 45.85±9.72 | ﹣6.704 | <0.001 |
| Vis | 42.75±11.78 | 49.14±9.03 | ﹣6.455 | <0.001 |
| RPS | 45.6±11.48 | 49.33±11.09 | ﹣3.566 | <0.001 |
| SC | 33.95±11.11 | 33.03±9.50 | 0.950 | <0.001 |
| IQ | 107.12±14.36 | 115.74±10.42 | ﹣7.253 | <0.001 |
| GAF | 37.12±4.53 | 55.45±8.39 | ﹣30.882 | <0.001 |

Data are mean ± standard deviation and T or Chi-square test.

SCH Schizophrenia, MOD Mood disorders, including major depressive disorder and bipolar disorder, SOP speed of processing, WM working memory, AV attention/vigilance, Vrbl verbal learning, Vis visual learning, RPS reasoning and problem solving, SC social cognition, IQ intelligence quotient, GAF Global Assessment of Functioning

# Supplementary Figures

***The network of cognitive and global function for all subjects***

Figure S1 Stability of network for all subjects


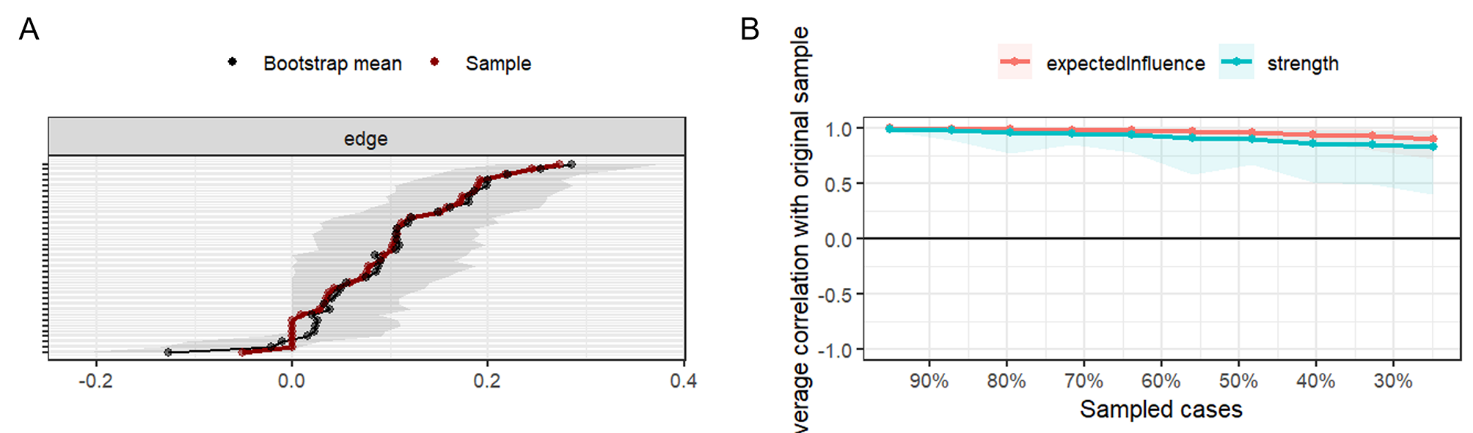


Note: A edge stability assessed by bootstrapping. The red line depicts the sample edge weights and the gray bar depicts the 95% confidence interval, edge-edge relationships are depicted on the y-axis with labels omitted. B stability of expected influence and strength. Correlations between expected influence and strength values in original sample and newly estimated expected influence and strength values during sample decline which are also called correlation stability coefficient (CS-C).

Figure S2 Centrality difference test (all the subjects)


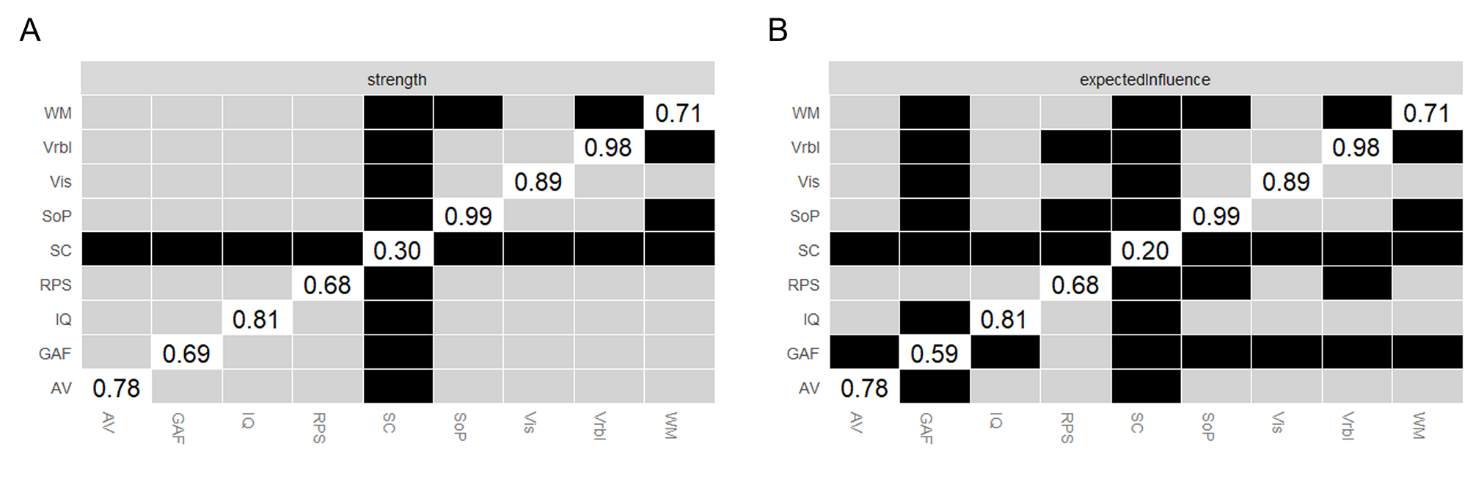


Note: A Centrality difference test of strength. B Centrality difference test of expected influence. Black squares represent significantly different centrality indexes. Grey squares represent non-significantly different centrality indexes.

***The network of cognitive and global function for different genders***

Figure S3 Stability of network for males

*
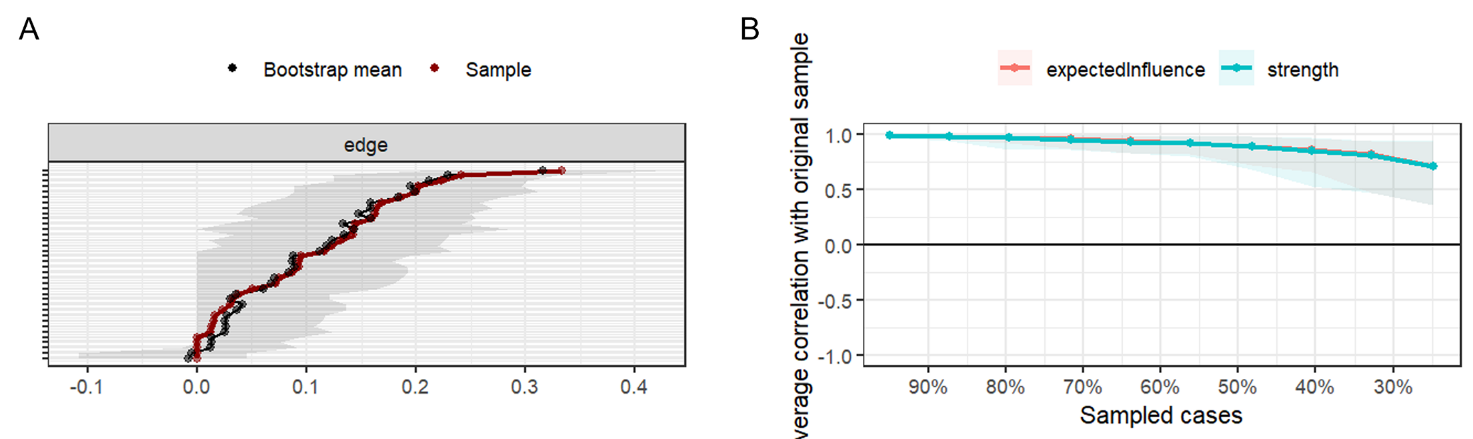
*

Note: A edge stability assessed by bootstrapping. The red line depicts the sample edge weights and the gray bar depicts the 95% confidence interval, edge-edge relationships are depicted on the y-axis with labels omitted. B stability of expected influence and strength. Correlations between expected influence and strength values in original sample and newly estimated expected influence and strength values during sample decline which are also called correlation stability coefficient (CS-C).

Figure S4 Centrality difference test (males)


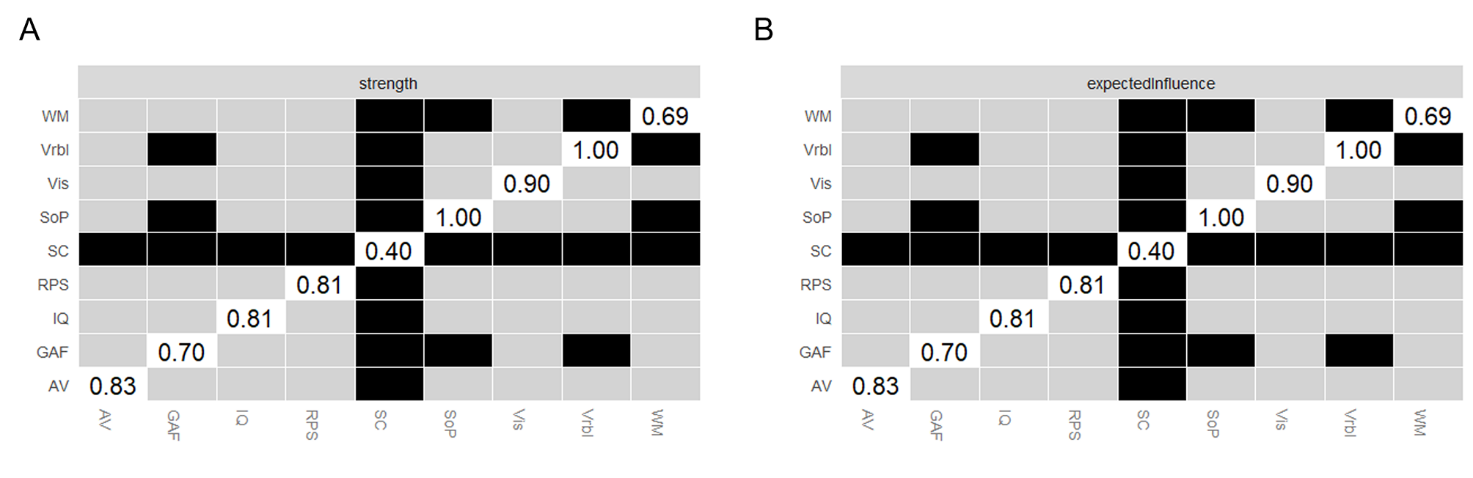


Note: A Centrality difference test of strength. B Centrality difference test of expected influence. Black squares represent significantly different centrality indexes. Grey squares represent non-significantly different centrality indexes.

Figure S5 Stability of network for females

*
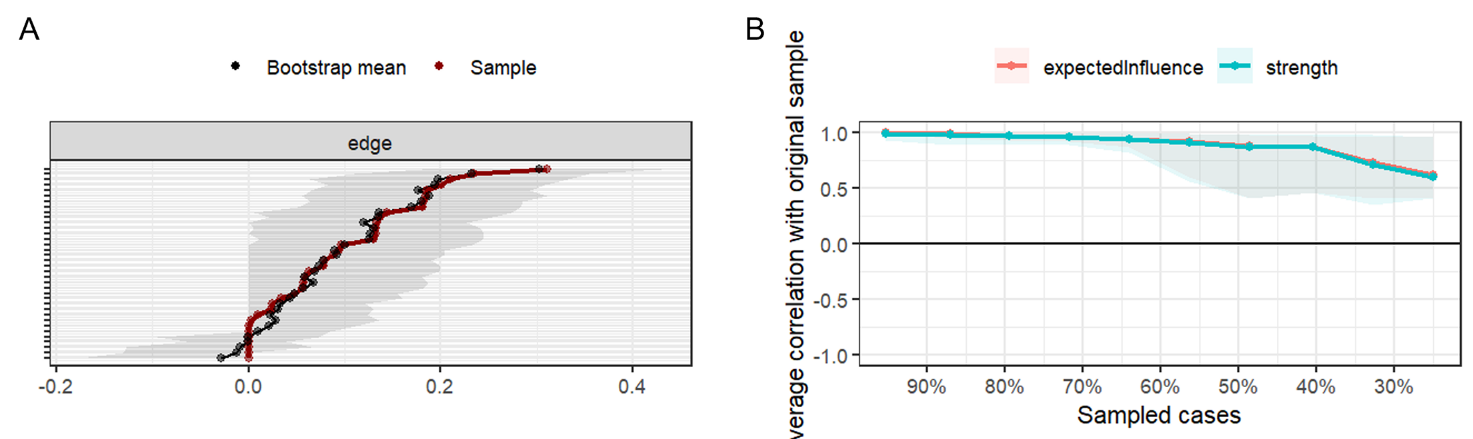
*

Note: A edge stability assessed by bootstrapping. The red line depicts the sample edge weights and the gray bar depicts the 95% confidence interval, edge-edge relationships are depicted on the y-axis with labels omitted. B stability of expected influence and strength. Correlations between expected influence and strength values in original sample and newly estimated expected influence and strength values during sample decline which are also called correlation stability coefficient (CS-C).

Figure S6 Centrality difference test (females)


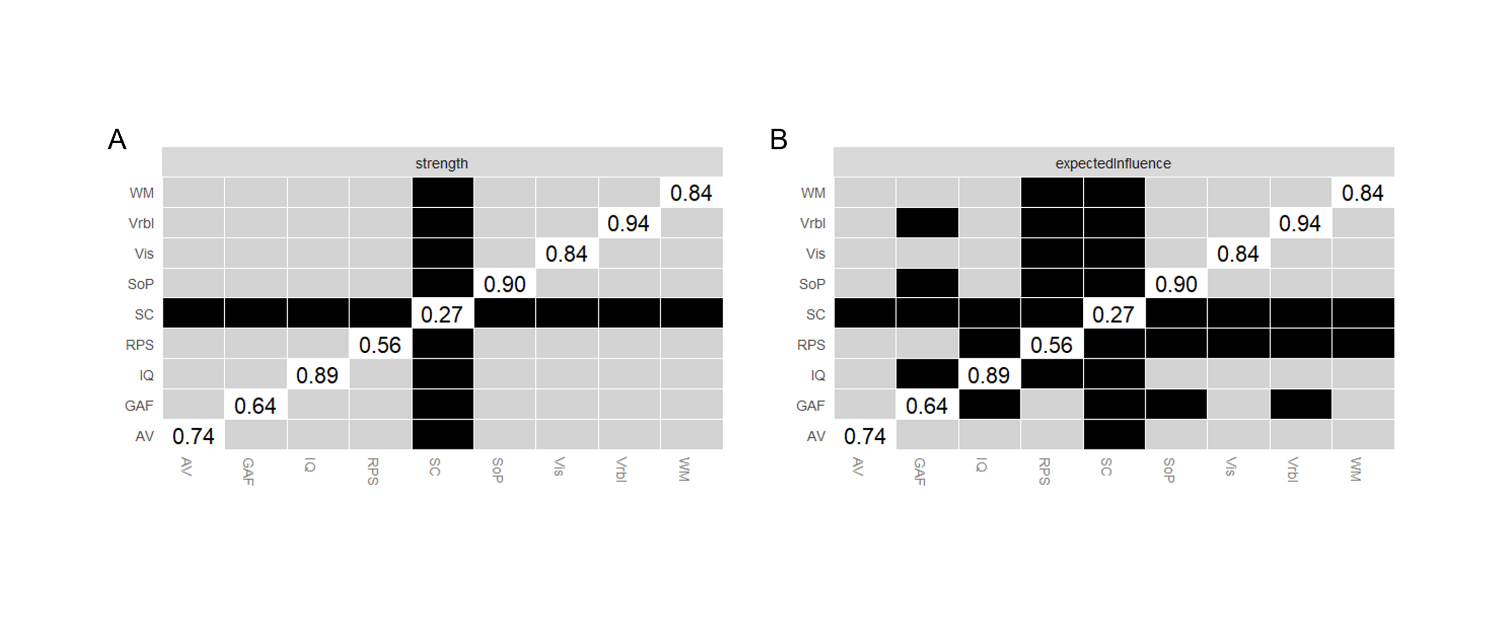


Note: A Centrality difference test of strength. B Centrality difference test of expected influence. Black squares represent significantly different centrality indexes. Grey squares represent non-significantly different centrality indexes.

***The network of cognitive and global function for Schizophrenia***

Figure S7 Stability of network for schizophrenia


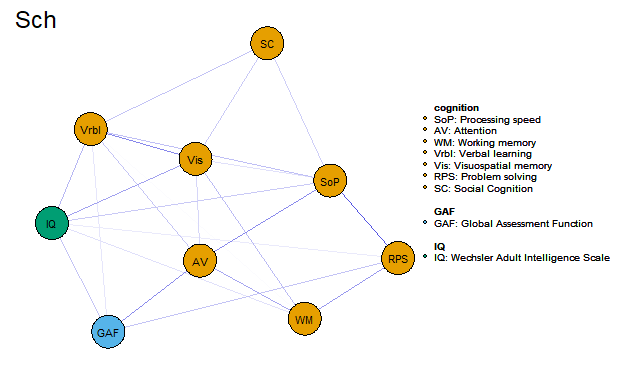


*
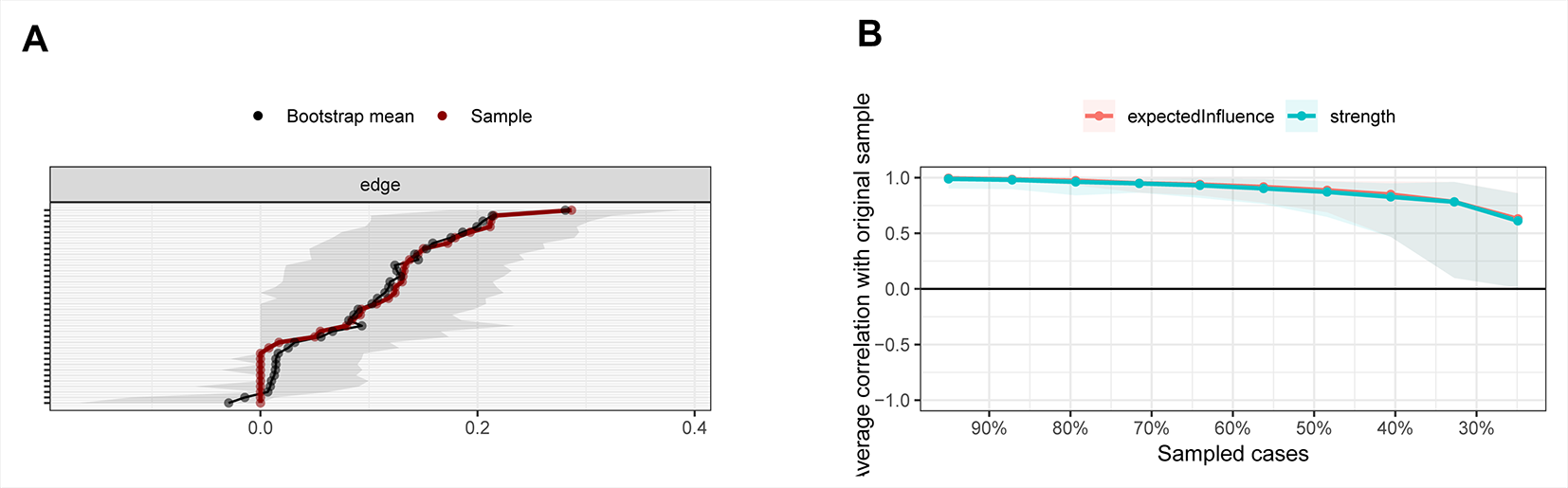
*

Note: A edge stability assessed by bootstrapping. The red line depicts the sample edge weights and the gray bar depicts the 95% confidence interval, edge-edge relationships are depicted on the y-axis with labels omitted. B stability of expected influence and strength. Correlations between expected influence and strength values in original sample and newly estimated expected influence and strength values during sample decline which are also called correlation stability coefficient (CS-C).

Figure S8 Centrality difference test (schizophrenia)


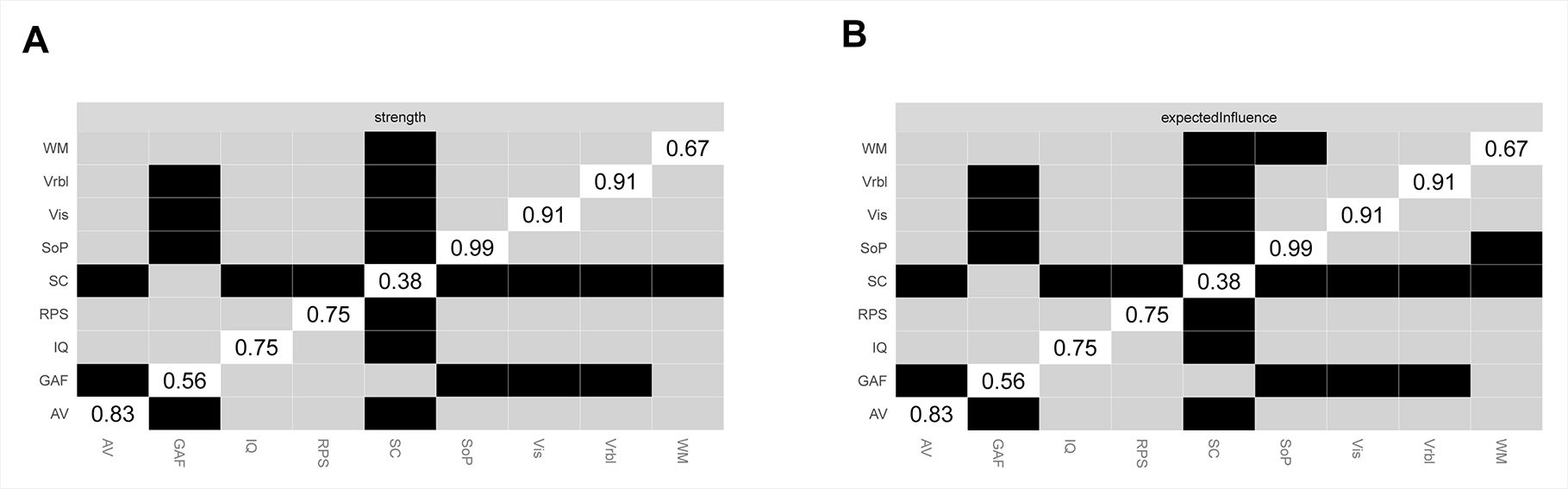


Note: A Centrality difference test of strength. B Centrality difference test of expected influence. Black squares represent significantly different centrality indexes. Grey squares represent non-significantly different centrality indexes.

Figure S9 The network of cognitive and global function for schizophrenia

Note: Blue edges represent positive associations whereas red edges represent negative associations, and thickness of an edge represents the strength of association between two nodes.

Figure S10 centrality of estimated network of the schizophrenia


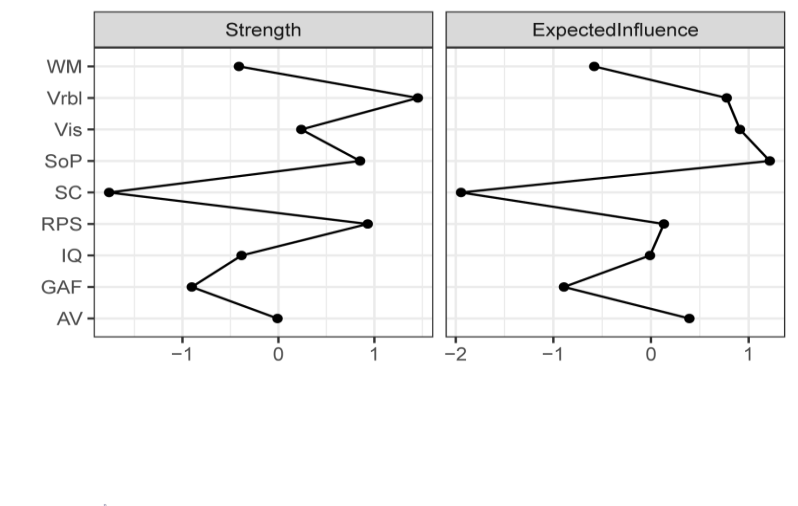


Note: Depicting the Strength and Expected influence of each node in schizophrenia. SOP speed of processing, WM working memory, AV attention/vigilance, Vrbl verbal learning, Vis visual learning, RPS reasoning and problem solving, SC social cognition, IQ intelligence quotient, GAF Global Assessment of Functioning.

**
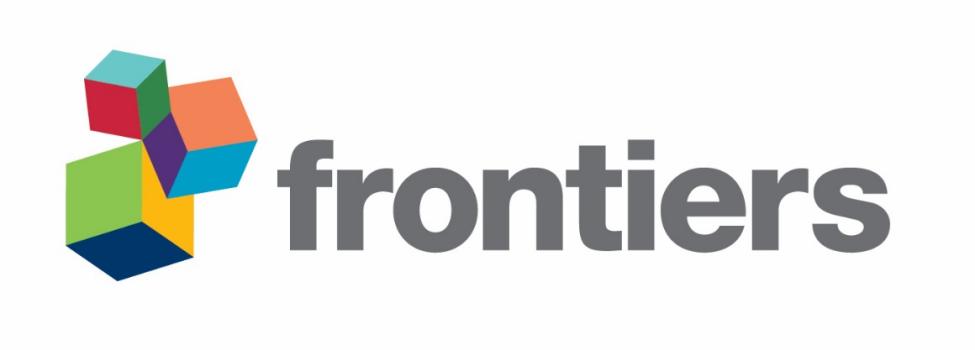
**
